# Hyperglycemia induced damage to mitochondrial respiration in renal mesangial and tubular cells: Implications for diabetic nephropathy

**DOI:** 10.1016/j.redox.2016.09.007

**Published:** 2016-09-17

**Authors:** Anna Czajka, Afshan N. Malik

**Affiliations:** Diabetes Research Group, Division of Diabetes and Nutritional Sciences, Faculty of Life Sciences and Medicine, King's College London, London, UK

**Keywords:** Cellular bioenergetics, Renal cells, Mesangial cells, Tubular cells, Mitochondrial dysfunction, Diabetes

## Abstract

Damage to renal tubular and mesangial cells is central to the development of diabetic nephropathy (DN), a complication of diabetes which can lead to renal failure. Mitochondria are the site of cellular respiration and produce energy in the form of ATP via oxidative phosphorylation, and mitochondrial dysfunction has been implicated in DN. Since the kidney is an organ with high bioenergetic needs, we postulated that hyperglycemia causes damage to renal mitochondria resulting in bioenergetic deficit. The bioenergetic profiles and the effect of hyperglycemia on cellular respiration of human primary mesangial (HMCs) and proximal tubular cells (HK-2) were compared in normoglycemic and hyperglycemic conditions using the seahorse bio-analyzer. In normoglycemia, HK-2 had significantly lower basal, ATP-linked and maximal respiration rates, and lower reserve capacity compared to HMCs. Hyperglycemia caused a down-regulation of all respiratory parameters within 4 days in HK-2 but not in HMCs. After 8 days of hyperglycemia, down-regulation of respiratory parameters persisted in tubular cells with compensatory up-regulated glycolysis. HMCs had reduced maximal respiration and reserve capacity at 8 days, and by 12 days had compromised mitochondrial respiration despite which they did not enhance glycolysis. These data suggest that diabetes is likely to lead to a cellular deficit in ATP production in both cell types, although with different sensitivities, and this mechanism could significantly contribute to the cellular damage seen in the diabetic kidney. Prevention of diabetes induced damage to renal mitochondrial respiration may be a novel therapeutic approach for the prevention/treatment of DN.

## Highlights

• The kidney as an organ has high bioenergetic needs.
• Cultured renal mesangial and tubular cells have different bioenergetic profiles.
• Hyperglycemia had a toxic effect on renal cell mitochondrial metabolism.
• Mesangial cells were more resistant to diabetes induced damage to cellular respiration than tubular cells.
• Energy deficit in renal cells may be a contributing factor to renal damage in diabetic nephropathy.

## Graphical abstract

**Figure:**
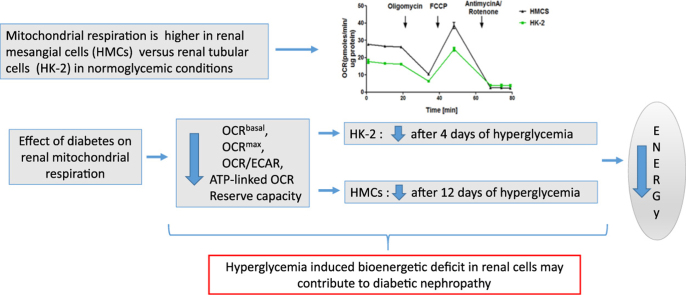

## 1 Introduction

Diabetic nephropathy (DN) is a kidney disease which affects approximately one-third of patients with diabetes and develops over a long period of clinical silence [1]. Despite good metabolic control, patients with both Type 1 and Type 2 diabetes are at risk of DN [2], [3], [4]. With the epidemic rise in the incidence of diabetes currently affecting more 350 million people worldwide, more than 100 million people are at risk of DN and approximately 30% of these are likely to progress to end stage renal failure despite therapy [5], [6]. Therefore, there is an urgent need to understand the underlying mechanisms that result in damage to the kidney in order to design novel preventative and therapeutic strategies.

DN is associated with structural changes in the kidney manifested as thickening of the glomerular basement membrane and expansion of the extracellular matrix (ECM) with deposition of collagen type IV and VI, fibronectin and laminin [7], resulting in glomerulosclerosis and tubular interstitial fibrosis, pathological hallmarks of DN. Mesangial cells are specialised smooth muscle cells located around the glomerular capillaries within the renal corpuscle of the kidney and have several functions, including the deposition of ECM. In DN, mesangial cells contribute to the disease by responding to increased glomerular filtration pressure by the expansion of the matrix into the capillary lumens and the secretion of pro-inflammatory cytokines [8], leading to glomerulosclerosis and glomerular inflammation [9]. Although, for a long time the major pathogenic mechanisms in DN were thought to stem from glomerulosclerosis through mesangial expansion, it is now widely accepted that tubular epithelial cells present in the lining of the nephrons are also involved and contribute to interstitial fibrosis, peritubular capillary loss and destruction of functioning nephrons due to tubular atrophy and severe tubulointerstitial lesions [10], [11], [12], [13].

Hyperglycemia is known to be a contributing factor in the development of DN through activation of a series of overlapping biochemical pathways [14]. Hyperglycemia-induced oxidative stress caused by the over-production of mitochondrial reactive oxygen species (ROS) has been implicated in this process [15], [16]. Indirect evidence for mitochondrial involvement in DN from animal and in-vitro studies has been accumulating [17], [18], [19], [20] and more recently, studies have directly demonstrated mitochondrial dysfunction in human clinical samples. For example Sharma et al., demonstrated that patients with DN had reduced urinary mitochondrial metabolites [21]. We recently reported that mitochondrial DNA and mitochondrial RNAs are altered in blood cells of DN patients, and in renal cells cultured in high glucose [22]. Despite the putative involvement of mitochondrial dysfunction in DN, the exact mechanisms involved remain unknown [17].

Kidneys are organs requiring large amounts of energy in form of ATP due to the re-absorption processes and although their mass accounts for less than 1% of total body mass, they use almost 10% of the body's oxygen which is utilized in cellular respiration [23]. Renal mRNA profiling shows one of the highest levels of mitochondrial transcripts, after colon, muscle and heart [24], suggesting an abundance of renal mitochondria. In support of this, we recently showed that mouse kidneys have one of the highest amount of MtDNA, second only to the heart, when compared to liver, lung, brain, islet and blood [25]. Eukaryotic cells, especially those with high energy demands like kidney cells, rely mostly on ATP generated through oxidative phosphorylation (OXPHOS), which is 15 times more productive than the amount produced by aerobic glycolysis [26]. OXPHOS takes place in the electron transport chain (ETC) located within the mitochondrial matrix and can be studied by precise measurement of oxygen consumption rate and proton leak, a process described as extracellular flux analysis, which can be used to define the bioenergetic properties of cells [27], [28], [29]. The use of specific inhibitors of various part of the ETC, which is made of 5 complexes of proteins, allows the definition of cellular bioenergetic profiles [30] and can provide information on cellular metabolism.

There have been very few studies of mitochondrial bioenergetics related to DN. We recently reported for the first time that peripheral blood mononuclear cells (PBMCs) from patients with DN have a severely diminished bioenergetic profile and reduced reserve capacity when compared to diabetes controls, the latter had a long duration of diabetes and no history of kidney disease [22]. This deficit could contribute to energy depletion, and indeed we found that mesangial cells grown in diabetic conditions show altered mitochondrial respiration [22]. Therefore, in the current study, we wished to test the hypothesis that diabetes can cause damage to mitochondrial respiration in kidney cells and to examine respiration in the two key cell types known to be implicated in DN, namely tubular and mesangial cells.

## 2 Materials and methods

### 2.1 Human mesangial and tubular cells

Primary human mesangial cells (HMCs) were cultured and characterised as previously described [31], [32] and grown in Dulbecco's Modified Eagle Medium (DMEM, Sigma Aldrich) supplemented with 10% FBS, ITS and antibiotics. Human cortex proximal tubular immortalized cells (HK-2, ATCC, LGC Standards) were cultured according to the manufacturer's conditions. Cells were seeded at an equal density (1×10^5^/well) in DMEM containing either 5 mM (normal, NG) or 25 mM (high, HG) glucose for 4 and 8 days. 20 mM mannitol in 5 mM glucose DMEM was used as an osmotic control in all experiments.

### 2.2 Extracellular flux analysis

The metabolic profiles of cultured HMCs and HK-2 cells were assessed using the XF^e^96 Seahorse analyser and XF cell mito stress test kit and XF-FluxPaks containing 96-well plates and cartridges (Agilent Technologies).

Oxygen consumption rate (OCR), a measurement of mitochondrial respiration, and extracellular acidification rate (ECAR), were determined in the presence of specific mitochondrial activators and inhibitors. Oligomycin (ATP synthase blocker) was used to measure ATP turnover and to determine proton leak. Mitochondrial un-coupler FCCP (carbonyl cyanide 4-[trifluoromethoxy] phenylhydrazone) was used to measure maximum respiratory function (maximal OCR). Reserve capacity was calculated as a maximal OCR minus the basal respiration. Rotenone (inhibitor of complex I) and antimycin A (a blocker of complex III), were injected to completely shut the mitochondrial respiration down, to confirm that any observed changes in respiration were mitochondrial [33], [34]. In order to determine optimal cell number and drug concentration, optimisation experiments with a different cell seeding number and titration of electron transport chain activators and inhibitors were carried out, to accurately assess OCR and ECAR as described by Dranka et al. [33].

One day prior to the extracellular flux measurement, HMCs and HK-2 cells were seeded at the concentration of 30,000 and 25,000 cells per well respectively in 96-well assay plates in complete growth medium containing 5 mm, 25 mM glucose and osmotic control. 1hr prior to the experiment the growth medium was removed, cells were washed 3 times and then incubated in low buffered assay medium (Agilent Technologies) supplemented with 5 mM glucose and 1 mM sodium pyruvate, pH 7.4. The microplates were then assayed in the XF^e^96 analyzer. For a measurement of steady basal respiration, 4–5 measurements were taken before injecting ATP synthase inhibitor, oligomycin at 1 μM (final concentration). Mitochondrial membrane uncoupler FCCP was then injected at 0.75 μM. Finally a mixture of rotenone and antimycin A (1 μM) was injected. Mitochondrial basal respiration, proton leak, spare capacity and maximal respiration were measured after correcting for non-mitochondrial respiration. After finishing the experiments 20 μl of cell lysis buffer [33] were added per well and protein content measured by a BCA assay (Fisher Scientific). OCR and ECAR rates were normalized to the protein content and presented as pmolesO_2_/min/μg protein and mpH/min/μg protein respectively.

### 2.3 Statistics

Analysis was performed using GraphPad (GraphPad Software, Inc). The distribution of the data was tested using the Kolmogorov-Smirnov test and histograms and parametric tests were used on raw data. Groups were compared using Student's *t*-test (2 groups) or one way ANOVA with post-hoc Tukey's multiple comparison test (>2 groups), statistical significance was considered at P<0.05. Data are presented as mean±SEM.

## 3 Results

### 3.1 Cellular bioenergetics of renal mesangial and tubular cells

**Fig. 1:**
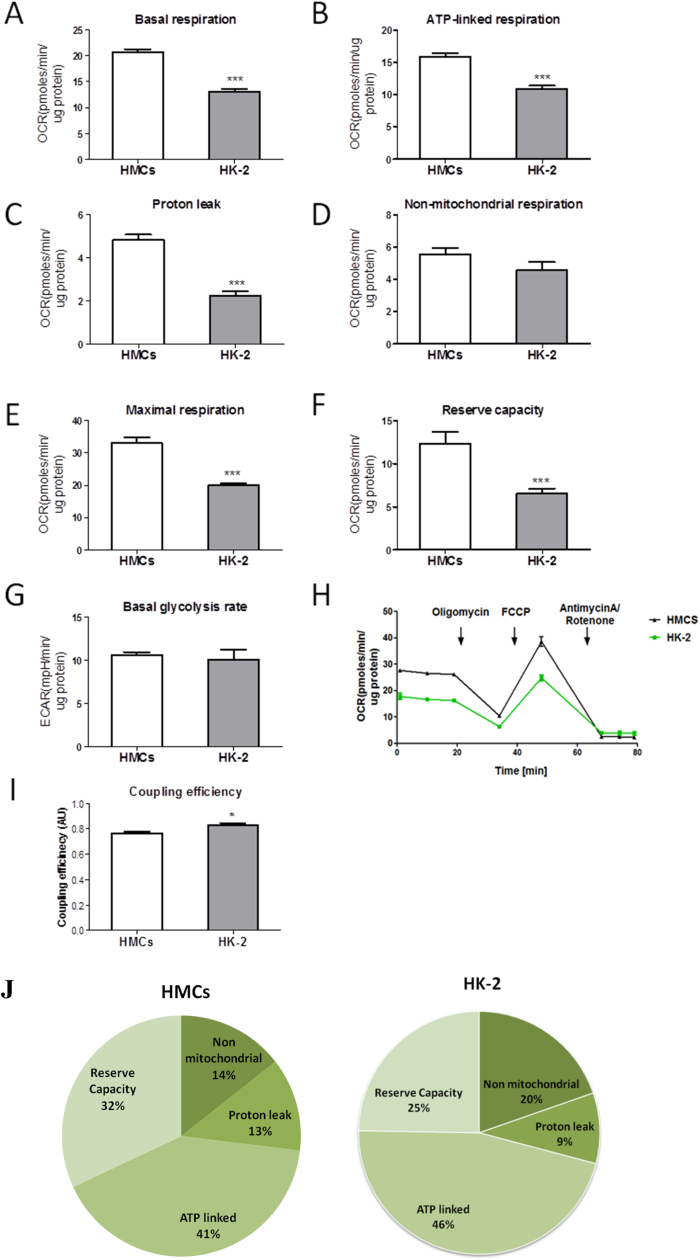
The bioenergetic profiles of human mesangial and tubular cells. Human glomerular mesangial (HMCs) and tubular (HK-2) cells were grown in DMEM containing 5mM glucose for 4 days and cellular bioenergetics was assessed: Four measurement of basal OCR were used to determine basal OCR (A); ATP- linked respiration (B) and proton leak (C) were determined following the injection of oligomycin (1 μM final); Maximal respiration (E) was determined after FCCP injection (0.3 μM); Reserve capacity was measured as the difference between maximal and basal respiration (F); Coupling efficiency (I); Bioenergetic profiles of HMCs and HK-2 with parameters expressed as a % of the maximal respiration following FCCP injection (J). All the parameters were calculated by subtracting non-mitochondrial values for each sample (D). Data shown as a mean ± SEM, n=7–14 replicates from 2 independent experiments. Independent student’s *t*-test where, **P<0.01, ***P<0.001.

We first characterised the bioenergetic profiles of mesangial (HMCs) and tubular (HK-2 cells) in normoglycemic conditions (5 mM glucose) using the seahorse XF^e^96analyser. Oxygen consumption rate (OCR) including basal, ATP-linked, maximal respiration and reserve capacity, and basal extracellular acidification rate (ECAR) which correlates to amount of protons released from the cell with potential contribution from glycolysis and the Krebs cycle were determined in real time (Fig. 1A-H) using specific activators and inhibitors of cellular respiration [30].

HK-2 cells had significantly lower (P<0.001) levels of basal respiration (13 pmolesO_2_/min/μg of protein) when compared to HMCs (20 pmolesO_2_/min/μg of protein, Fig. 1A, 1H). Following the injection of oligomycin, an ATP synthase inhibitor, basal OCR is expected to drop and the level of drop can be used to deduce ATP-production (ATP-linked respiration). ATP linked respiration was significantly lower (P<0.001) in HK-2 cells (11 pmolesO_2_/min/μg protein) compared to HMCs (16 pmolesO_2_/min/μg of protein, Fig. 1B, 1H). Injection of mitochondrial uncoupler, FCCP caused an increase in OCR and allowed measurement of the maximal mitochondrial respiration and reserved capacity. Maximal OCR was significantly lower (P<0.01) in HK-2 cells (20 pmolesO_2_/min/μg of protein) compared to HMCs (33 pmolesO_2_/min/μg of protein, Fig. 1E, 1H). Reserve capacity was also significantly lower (P<0.01) in HK-2 cells (6.5 pmolesO_2_/min/μg of protein) compared to HMCs (12 pmolesO_2_/min/μg of protein, Fig. 1F).

Proton leak, measured as the difference between ATP-linked and basal OCR, was significantly lower (P<0.001) in HK-2 cells (2.2 pmolesO_2_/min/μg of protein) compared to HMCs (4.5 pmolesO_2_/min/μg of protein, Fig. 1C). Non-mitochondrial respiration, measured as a drop in OCR following the administration of mitochondrial respiratory chain blockers, rotenone and antimycin A, did not vary significantly between the two cell types, and was measured as 4.6 and 5.6 pmolesO_2_/min/μg of protein for HK-2 and HMCs respectively (P>0.05, Fig. 1D). Basal glycolytic rates were also measured at similar levels between both experimental cell types (P>0.05, Fig. 1G).

We next evaluated the coupling efficiency defined as a proportion of mitochondrial oxygen consumption used for the ATP synthesis (Fig. 1I). Coupling efficiency represents the proportion of the substrate oxidation energy used for the ATP production, while the rest is lost as a heat. Of all the oxygen consumed, HK-2 cells use a significantly higher rate for ATP-linked respiration relative to HMCs (P<0.05, 0.8 versus 0.75 respectively, Fig. 1I).

The proportional distribution of oxygen consumption and utilization among the different parameters defined in the bioenergetic profile illustrate the higher reserve capacity but lower ATP-linked respiration in HMCs compared to HK-2 cells (Fig. 1J).

### 3.2 Hyperglycemia induced alterations in cellular respiration in renal tubular cells

**Fig. 2:**
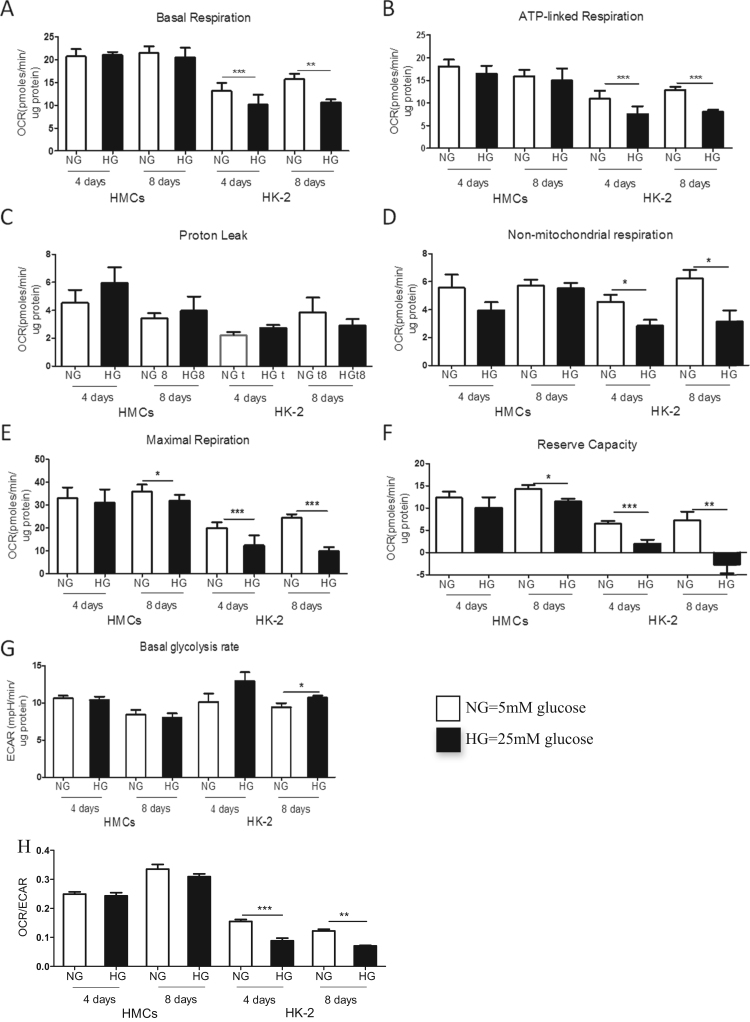
Bioenergetic profile of human kidney cells exposed to hyperglycemia. Human mesangial (HMCs) and tubular (HK-2) cells were grown in DMEM containing normal (5 mM) glucose (NG), and high (25 mM) glucose (HG), and in osmolarity controls using mannitol (data not shown) for 4 and 8 days. One day prior to the assessment, cells were trypsinised, counted and seeded at density of 25-30,000 cells/well. Oxygen consumption rate (OCR) was assessed using the Seahorse analyser: Four measurement of basal OCR were used to determine basal OCR (A); ATP- linked respiration (B) and proton leak (C) were measured after oligomycin injection (1 μM final concentration); Maximal respiration (E) was determined after FCCP injection (0.3 μM); Reserve capacity was the difference between maximal and basal respiration (F); Extracellular acidification rate (ECAR) representing glycolysis is shown on figure G; Metabolic rates were expressed as the ratio of basal OCR and ECAR (H). All parameters were calculated by subtracting non-mitochondrial values for each sample (D). Data shown as a mean ± SEM, n=7–17 replicates from 2 independent experiments. Independent student’s *t*-test where, *P<0.05, ***P<0.001.

The effect of hyperglycemia on cellular respiration in HK-2 cells was examined. Growth in high glucose caused a significant down-regulation of all respiratory parameters. Basal, ATP-linked and maximal respiration as well as reserve capacity were reduced within four days (P<0.01) and remained reduced after prolonged exposure over 8 days (Fig. 2A, B, E, F). No significant changes were detected in proton leak (P>0.05, Fig. 2C), but non-mitochondrial respiration was significantly reduced following growth in HG (P<0.05, Fig. 2D). Basal ECAR was significantly up-regulated after 8 days growth in HG (P<0.05, Fig. 2G).

HK-2 cells had significantly down regulated metabolic rate (plotted as a ration of OCR and ECAR) when cultured in HG for 4 and 8 days and compared to NG controls (P<0.001, P<0.01 respectively Fig. 2I). Mitochondrial respiration was unaltered in osmolarity controls and was unaffected by the duration of culturing time (Supplementary Fig. 1 and Fig. 2) showing that the changes in respiration of tubular cells were a direct consequence of growth in hyperglycemia.

### 3.3 Hyperglycemia induced changes in cellular respiration in renal mesangial cells

Unlike tubular cells, growth of mesangial cells in HG for 4 days did not affect their bioenergetic profiles, there were no significant changes in basal, ATP-linked, maximal respiration, and basal glycolytic rate remained unchanged (P>0,05, Fig. 2A-E). Growth of mesangial cells in HG for 8 days still did not affect basal and ATP-linked respiration and basal glycolytic rate (P>0.05), however maximal OCR and reserve capacity were reduced (P<0.05, Fig. 2A-E and G). No statistically significant difference was detected in the non-mitochondrial respiration (P>0.05, Fig. 2F) and in basal glycolytic rate (P>0.05, Fig. 2H).

The metabolic rates of mesangial cells were plotted as the ratio of OCR and ECAR under basal conditions and were unaltered in hyperglycemic conditions (P>0.05, Fig. 2I). Mitochondrial respiration was unaltered in osmolarity controls and was unaffected by duration of culturing time of mesangial cells (Supplementary Fig. 1 and Fig. 2).

**Fig. 3:**
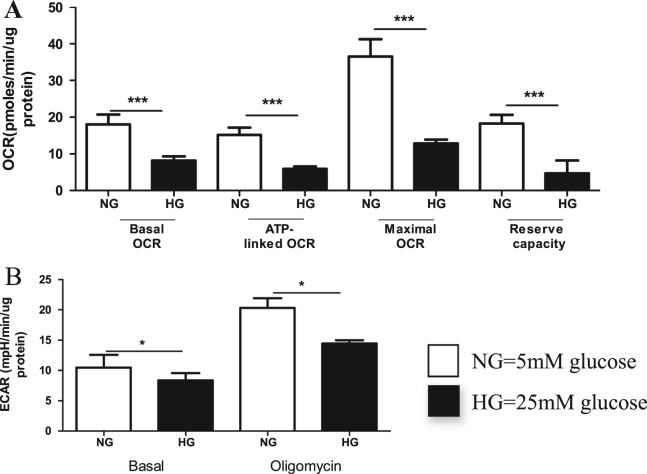
Prolonged hyperglycemia affects all bioenergetics parameters of human mesangial cells. HMCs were grown in 5 mM (NG), 25 mM (HG), and osmolarity controls (not shown) for 12 days. The day before the assessment, cells were trypsinized, counted and seeded at density of 35,000 cells/well, and oxygen consumption rates (OCR) were assessed in the Seahorse analyzer. (A) Four measurement of basal OCR were used to determine basal OCR; ATP- linked respiration and proton leak were measured after oligomycin injection (1 μM) and maximal respiration was determined after FCCP injection (0.3 μM); Reserve capacity was the difference between maximal and basal respiration; (B) Extracellular acidification rates (ECAR) were used to represent glycolysis and basal ECAR and ECAR after injection of the oligomycin (1 μM final) are shown. Values shown are mean ± SD, n=8 observations, Independent student’s *t*-test where, *P<0.05.

**Fig. 4:**
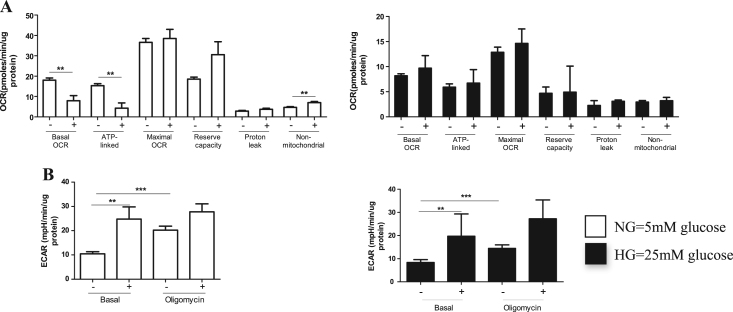
Prolonged exposure to hyperglycemia affects the bioenergetic response to acute stress in mesangial cells. HMCs were grown in 5 mM (NG), 25 mM (HG) or osmolarity controls for 12 days. One day prior to the assessment, cells were seeded at density of 35 000 cells/well in assay medium. Acute stress was induced by injection of 20 mM glucose (+) or normal assay media (-) during the assessment in Seahorse analyzer. Oxygen consumption rates (OCR) (A) and extracellular acidification rates (ECAR) (B) are shown for cells which has been grown in normoglycemia (left panel) and cells which had been grown in hyperglycemic conditions (right panel) for 12 days. Data shown are mean values ± SEM, n=4-8 observations, Student’s *t*-test where, ** P<0.01, ***P<0.001.

Since HMCs seemed more resistant to hyperglycemia than HK-2 cells, we decided to determine the effect of longer exposure to hyperglycemia, therefore HMCs were cultured for 12 days in normal and HG (Fig. 3). 12-d exposure to HG decreased basal oxygen consumption rate in HMCs by more than 50% when compared to control (P<0.0001, Fig. 3A) and the respiration used for the ATP production reduced by 2.5 fold when compared to cells cultured in NG (P<0.0001, Fig. 3A). There was no difference in proton leak and in non-mitochondrial respiration (P>0.05, data not shown). As described above, maximal respiratory capacity of cells, was significantly lower in the cells cultured for 8 days in HG when compared to the controls, and this reduction became highly significant (P<0.0001) after 12 days of culture in HG, with 3 fold reduction of maximal OCR (P<0.0001, Fig. 3A) and 4 fold reduction in the reserve capacity when compared to cells cultured in NG (P<0.001). When glycolysis was assessed in these cells, there was also a significant reduction in glycolysis at basal level (P<0.05) and also after the addition of oligomycin (which stimulates glycolysis due to inhibition of ATP synthesis, P<0.01, Fig. 3B). Furthermore, we found that the ability of cells to respond to acute stress was also blunted after 12 days growth in hyperglycemic conditions (Fig. 4). HMCs grown in normoglycemic conditions for 12 days were able to respond to acute stress by altering their metabolism, whereas the hyperglycemic cells had lost this ability (Fig. 4). After acute glucose load, both normoglycemic and hyperglycemia HMCs switched their metabolism to glycolysis (Fig. 4), however hyperglycemic HMCs which were previously exposed to chronic hyperglycemia had lost their flexibility to respond to nutrient load, as they could not increase their glycolytic rate but at the same time mitochondrial respiration remained the same which suggested they cannot balance their energy production.

In summary, we showed that 4 days exposure to hyperglycemia had no effect on any of the measured parameters involved in mitochondrial respiration or glycolysis in HMCs, 8 days exposure significantly decreased maximal respiration and reserve capacity but did not affect any other mitochondrial respiration parameters, whereas 12 days exposure significantly diminished both mitochondrial respiration and glycolytic capacity, suggesting an energy deficit in these cells.

## 4 Discussion

With the global rise in the incidence of diabetes, diabetic nephropathy, a major risk factor for morbidity and mortality, is likely to reach epidemic levels in the next decade. To combat this, there is a strong clinical need to understand the early molecular mechanisms that lead to kidney damage in diabetes. The kidney as an organ has a high requirement for energy in the form of ATP derived from cellular respiration to drive cellular processes [23], [24]. Pathological changes in renal mesangial and tubular cells are well known to play significant roles in the development and progression of DN and lead to glomerulosclerosis and tubulointerstitial fibrosis [10], [12], both structural hallmarks of DN, but the role of energy deficit in these processes has been largely unaddressed in the past. Mitochondrial dysfunction in the diabetic kidney disease is increasingly being seen as a key mechanism in DN [21], [22] leading us to speculate that damage to the cellular respiratory apparatus in the diabetic kidney could lead to energy deficit and cellular damage. In order to shed light on the bioenergetic properties of renal cells, in the current paper we sought to define the bioenergetic profiles of renal mesangial and tubular cells and to assess whether diabetes can damage cellular respiration in these cells.

Cells need the ability to produce sufficient energy to carry out their normal cellular function, and have differing bioenergetic requirements depending on their function. In addition, cells also have to be able to respond to stressors by appropriately altering their metabolism. Our data show that both HMCs and HK-2 cells have distinct metabolic profiles and both cell types utilize more than two thirds of the consumed oxygen into ATP production. HMCs’ mitochondrial respiratory parameters are much higher when compared to HK-2 cells in normoglycemia, but tubular cells’ coupling efficiency is significantly higher, meaning that of all the oxygen consumed, HK-2 cells use a slightly higher proportion for ATP-linked respiration relative to HMCs and lose less energy through proton leak.

The bioenergetic profile of both mesangial and tubular cells was negatively affected by exposure to hyperglycemia but tubular cells were more susceptible, with cellular respiration parameters being significantly altered within 4 days of exposure to high glucose, and showing severe damage to cellular respiration by 8 days. In contrast, mesangial cells showed little change in high glucose even after 8 days. After prolonged high glucose exposure of 12 days, mesangial cells showed signs of decreased mitochondrial respiratory function, reduced their reserve capacity. Hyperglycemia had no effect on the proton leak in HMCs and HK-2, but tubular cells had decreased non-mitochondrial respiration when exposed to high glucose. Therefore our data show that cellular respiration is reduced in renal cells after exposure to hyperglycemia, and that mesangial cells are more resistant to this damage than tubular cells but do become damaged and more sensitive to acute stress after prolonged exposure.

Whilst mitochondrial and glycolytic metabolism have been proposed to be altered in metabolic disease [35], few studies have examined the effect of hyperglycemia on these parameters. Hyperglycemia has been previously shown to have varying effects on bioenergetics and cellular respiration in cultured cells, including decreased, unaltered and increased cellular respiration. For example, a decline in mitochondrial basal and maximal respiration was seen in rat retinal endothelial cells after 3 and 6 days of culture [36], and down-regulation of mitochondrial function (including reduced ATP-linked respiration and reserve capacity) was seen in primary cultured mouse mesangial cells exposed to hyperglycemia for 14 days [38], in human mesangial cells after 8 days [22]. A faulty bioenergetic profile was observed in cultured neurons derived from streptozotocin induced diabetic rats with decreased maximal respiration and decreased spare capacity [39]. No changes in respiration were detected in bovine aortic endothelial cells after 24 h exposure to hyperglycemia and in human platelets cultured in high glucose for 18 h [37], interestingly these studies had shorter duration of exposure to hyperglycemia than those that observed decreases. In contrast, Stieger at al., (2012) demonstrated an increase in the basal and maximal respiration of immortalized mouse podocytes cultured in high glucose for 10 passages. [40]. Measurement of cellular respiration directly in live human blood cells from patients with diabetes has also been attempted by us and others. Increased mitochondrial basal, and maximal respiration rates were found in peripheral blood mononuclear cells (PBMCs) from patients with type 2 diabetes (T2D), when compared to healthy controls [41]. We previously found a slight but non-significant increase in cellular respiration in PBMCs from diabetes patients without complications (both T1D and T2D) compared to healthy controls, however we found that PBMCs of patients with DN had reduced reserve capacity and increased sensitivity to stress, showing loss of metabolic flexibility [22]. Mitochondrial respiration was also reported to be reduced in the skeletal muscle of T2D patients [42], [43] and not changed in myocytes from non-diabetic, obese subjects with family history of T2D [44].

Using isolated mitochondria from diabetic models, Ruggiero at al., (2011) reported increased levels of mitochondrial superoxide dismutase (MnSOD) and increased mitochondrial ATP-linked respiration in 12 week old diabetic animals, but this increase was not observed at later stages, [45]. This suggests an adaptive response in mitochondrial respiration in order to cope with nutrient overload, and an increase in maximal and basal respiration which, when within threshold limit for mitochondria without diminishing spare respiratory capacity may be still tolerable. Longer exposure hyperglycemia might be detrimental as we have previously demonstrated that hyperglycemia can also damage MtDNA which could affect mitochondrial function over time [22]. If there is an adaptive phase when mitochondria increase their respiration in response to nutrient overload, then this could contribute to increased ROS production and further exacerbate cellular stress. Anderson at al., (2009) reported increased mitochondrial H_2_O_2_ production in human and rodent muscles fed with high fat diets, which correlated with a shift in cellular redox balance, without any changes in mitochondrial respiration. The oxidation state of the cells was balanced following treatment with mitochondria-specific antioxidants [46].

The data from the current study support the view that cells have different tolerance levels for chronic hyperglycemia which may lead to defective mitochondria that cannot properly respond to changes in glucose levels, and this could lead to possible disruption in glucose metabolism. HK-2 cells increased their basal ECAR in response to high glucose, which may be a compensatory response to the condition when cells cannot rely on their OXPHOS energy, but try to keep balance in the cellular ATP production [36], [47], or it could be caused by negative regulation of the OXPHOS through glycolytic intermediates [48]. However, when the ratio of both OCR and ECAR were compared, tubular cells seem to be metabolically less active in the presence of hyperglycemia when compared to the mesangial cells after 1 week of culture.

Energy production in the kidneys, which are mitochondrial rich organs, is largely through respiration, and Warburg demonstrated that the total energy production in quiescent kidney cells was similar to that produced in proliferating cancer cells, which indicates how much energy the kidney requires for proper functioning [49]. In support of this, we found that after the heart, kidney has the second highest mitochondrial DNA content when compared to other mouse organs, including liver, blood, brain, islets and lungs [25] suggesting that kidneys are rich in mitochondrial content. The data shown in the current study clearly show that high glucose had a toxic effect on renal cell mitochondrial metabolism, but the mechanisms by which this was caused remains unclear. Reserve capacity, described as a mitochondrial bioenergetic reserve, was significantly reduced in hyperglycemia in both mesangial and tubular cells. Reserve capacity depends on many factors, like substrate supply, energy demand and integrity of the electron transport chain. Dysfunction in mitochondrial metabolism could be caused by the direct effect of the glucose through modification of mitochondria-localised proteins, as described by others [50], [51], [52], [53]. But it could also be caused by the disruption in the synthesis of mitochondrial complexes assembly subunits, caused by mutations in MtDNA. We have previously detected a clear difference in metabolic function between the patients with diabetes without and with DN and we have shown that growth of renal cells in high glucose can rapidly change the amount of MtDNA and cause MtDNA mutations, leading to the proposal that the inability to cope with energy demand due to MtDNA damage could be a key factor in the progression of DN [22]. If systemic mitochondrial dysfunction is associated with DN, then we would predict systemic changes in the body which may be the basis of renal microvascular complications in diabetes. In such a scenario, different cells in the body would show different levels of sensitivity to hyperglycemia induced damage, the extent of which would be related to but not exclusive to mitochondrial content and bioenergetic need.

## 5 Conclusion

In conclusion, we have shown that hyperglycemia alters the metabolic profile of both mesangial and tubular cells by reducing their mitochondrial respiratory function. Mesangial cells have a substantial bioenergetic reserve compared to tubular cells. These results show that distinct cells within one organ can have different bioenergetic profiles and sensitivities to hyperglycemia. A thorough understanding of the bioenergetic properties of different cell types to oxidative stress and nutrient overload, present in diabetes and its complications is needed and could lead to the development of novel therapies to protect mitochondrial respiration in organs with high energy requirements.
