# Supplementary material for: Hyperglycemia induced damage to mitochondrial respiration in renal mesangial and tubular cells: Implications for diabetic nephropathy
Source: Redox Biol. 2016 Sep 17;10:100–7. doi: 10.1016/j.redox.2016.09.007 (PMC5053113; doi:10.1016/j.redox.2016.09.007)
Supplement: Supplementary file 1 — Supplementary material [file mmc1.docx]

**Supplementary data**

**Fig.S1.** **Protein content in renal cells.**

NG=5mM glucose

HG=25mM glucose

**Fig.S2 Bioenergetic profile of human kidney cells cultured in control conditions: osmolarity controls show no effect**

**
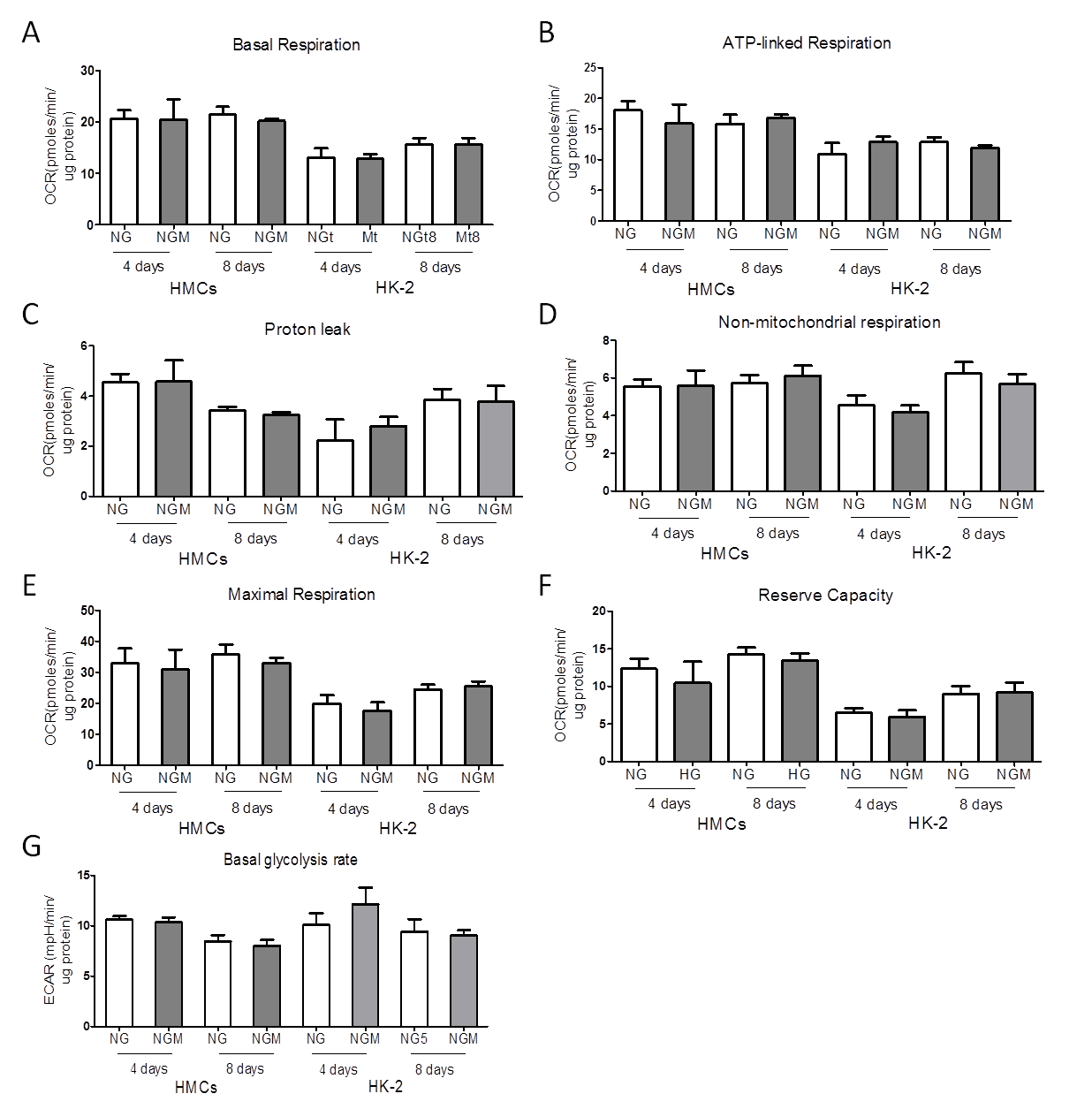
**

NG=5mM glucose

NGM=5mM glucose +20mM mannitol

HG=25mM glucose
